# Ionic Transport Triggered by Asymmetric Illumination on 2D Nano-Membrane

**DOI:** 10.3390/molecules26237078

**Published:** 2021-11-23

**Authors:** Linhan Du, Xiaoyu Hu, Diannan Lu, Zheng Liu

**Affiliations:** State Key Laboratory of Chemical Engineering, Department of Chemical Engineering, Tsinghua University, Beijing 100084, China; dulh20@mails.tsinghua.edu.cn (L.D.); xy-hu15@mails.tsinghua.edu.cn (X.H.); liuzheng@mail.tsinghua.edu.cn (Z.L.)

**Keywords:** nanochannel, MD simulation, ion sieving, asymmetric illumination

## Abstract

Ionic transport and ion sieving are important in the field of separation science and engineering. Based on the rapid development of nanomaterials and nano-devices, more and more phenomena occur on the nanoscale devices in the field of thermology, optics, mechanics, etc. Recently, we experimentally observed a novel ion transport phenomenon in nanostructured graphene oxide membrane (GOM) under asymmetric illumination. We first build a light-induced carriers’ diffusion model based on our previous experimental results. This model can reveal the light-induced ion transport mechanism and predict the carriers’ diffusion behavior under different operational situations and material characters. The voltage difference increases with the rise of illuminate asymmetry, photoresponsivity, recombination coefficient, and carriers’ diffusion coefficient ratio. Finally, we discuss the ion transport behavior with different surface charge densities using MD simulation. Moderate surface charge decreases the ion transport with the same type of charge due to the electrostatic repulsion; however, excess surface charge blocks both cation and anion because a thicker electrical double layer decreases effective channel height. Research here provides referenced operational and material conditions to obtain a greater voltage difference between the membrane sides. Also, the mechanism of ion transport and ion sieving can guide us to modify membrane material according to different aims.

## 1. Introduction

Nanofluidic is generally defined as the flow phenomenon in 1 to 100 nanometers on at least one dimension [1]. The nanoscale world shows excellent characteristics due to the scale effect, such as surface-energy-related, shear-related, and electrical double-layer-related phenomena [2], which promote the appearance of DNAs count and measure meter [3], concentration difference converts to electric energy devices [4], and nanofluidics diode or transistor [5,6]. Besides, these nanoscale phenomena are fundamental for many natural applications in mechanics, thermodynamics, electrokinetics, and biophysics [7,8,9]. For example, nanofluidic can play an important role in biological systems, such as biological water channels (aquaporins) [10] and ionic pumps (in nephrons and neurons) [11,12]. The unique nanostructure of aquaporins, asparagine-proline-alanine NPA motif pattern, only allows water molecules to go through the membrane in the right way efficiently. Therefore, the design and fabrication of novel materials with nanochannel attract more attention in recent years [13]. Nowadays, ion transport phenomena in nano-channel have attracted considerable attention because an ionic channel is utilized in a nanopore device, which enable us to detect coronavirus [14] and exosome [15]. For example, researchers canWe can also design and fabricate different nanostructured materials to realize heat driving active ion transport [16], cell identification and sorting [17], and light to electricity conversion [18].

Recent years witness the boost of 2D nanomaterials [19,20,21], such as graphene, graphene oxide, reduced graphene oxide, h-BN, MoS2, etc. By doping with metal or nonmetal elements, more kinds of nanomaterials have been designed and fabricated. Graphene oxide is one of the typical materials used to fabricate GO membrane with nanochannel structures [22]. These nanostructured membranes have already been used in filtration [23], desalination [24], ion trapper [25] and energy harvesting [26]. Besides, GO membrane has tremendous potential on anhydrous proton transfer devices [27], medical instruments [28], and high performance nanocomposites [29]. In 2013, Huang et al. [30] reported a nanostrand-channelled graphene oxide ultrafiltration membrane with a network of nanochannels. It has a narrow size distribution (3–5 nm) and superior separation performance. Wang et al. [21] studied metal oxide or metal ions as multifunctional cross-linkers to construct fast and carbon dioxide-selective nanochannels in few-layered GO membranes, which exhibit high separation performance. By mimicking membrane protein on their function of transport and catalysis [31], researchers fabricated high-efficiency devices on the electrochemical device [32], heterogeneous catalysis [33], superhydrophobic materials synthesis [34], and targeted drug delivery [35]. In our previous work, an exceptional ionic and molecular transport property has been discovered in layered graphene oxide membranes with sub-nanometer-wide lamellar channels [36]. We used vacuum filtration to fabricate the graphene oxide membrane with a 1.26 nm diameter pipe (bibulous) and discovered a synchronous photo-electric response in different electrolyte solutions.

Most importantly, when the light was illuminated asymmetrically on the membrane, the anti-concentration gradient ionic moving was realized depending on both light intensity and ion concentrations [36]. Based on the carriers’ diffusion model, we established a plausible mechanism to illustrate this phenomenon. Following the mechanism, photo-induced ion transport has great potential on active ion sieving and artificial photosynthesis on synthetic nanofluidic circuits, such as photonic ion switches, photonic ion diodes, and photonic ion transistors. However, our previous work only explains the phenomenon under the given parameters and situation. To employ this material more widely, we test its performance under different operational conditions and understand how it could be affected by different materials.

Here, we employ a carriers’ diffusion model to describe the charge distribution and electric potential difference between two ends based on standard dry graphene oxide membrane (GOM) with asymmetric light illumination on millimeter scale. Then we optimize the character parameters, trying to find out the relationship between the parameter and electric potential difference and the most efficient parameter group for voltage difference along the GOM (the driven electric field). Finally, we conduct the MD simulation by NAMD (NAnoscale Molecular Dynamics) to estimate the photocurrent at different nanochannel surface charge densities and different driven electric fields, illustrating the process of active ion sieving.

## 2. Models and Methods

**Figure 1:**
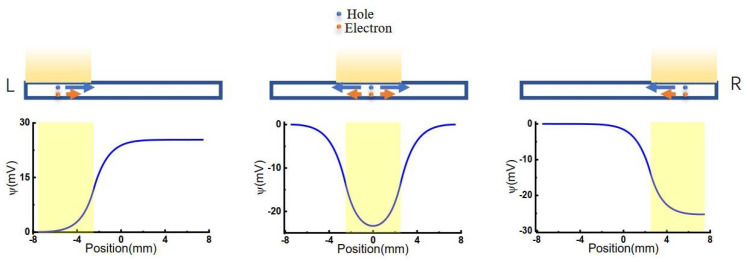
Scheme of carriers’ diffusion and electromigration.

**Figure 2:**
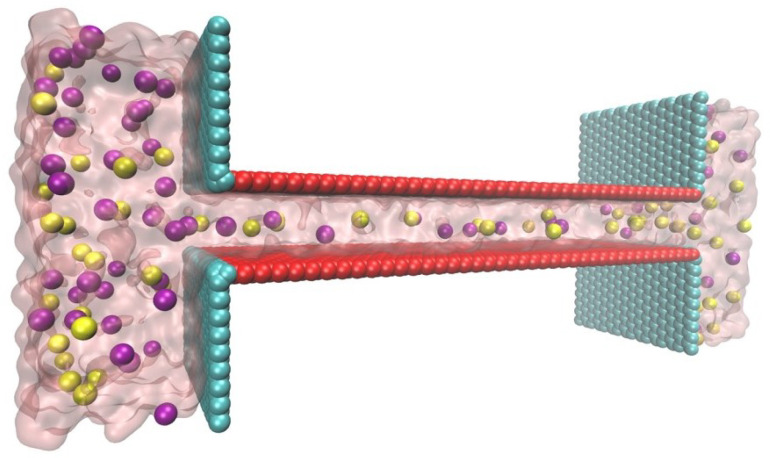
Schematic of the MD simulation structure drawn by VMD [44]. $K^{+}$ and ${\mathrm{Cl}}^{-}$ ions are in purple and yellow, respectively. The charged surface is colored red and the neutral wall is cyan.

The whole study on light-induced ion transport is divided into two parts. First is the voltage difference generation due to the light-induced carriers (hole-electron) decomposition, which is the driven force on ions in the channel (electric osmosis force, from L to R, Figure 1 and along the horizontal channel, Figure 2 and the “length” in channel size). Secondly, the voltage difference is transferred into an electric field and is set in molecular simulation. Meanwhile, the surface charge is to mimic the real graphene oxide surface charge, which usually is negative and can be changed with doping. Also, this part is to test how nanochannel material influences ion transport under the same driven force.

### 2.1. Carriers’ Diffusion Model

When light is illuminated on the membrane, excitons are generated firstly [37], depending on light and wavelength intensity. Then, excitons (also electron-hole pair) will split into electrons and holes [38]. Here, we focus on recombination and diffusion of holes and electrons after photo-illumination. Thus, the conceptional reaction equation can be written as $electron∼holepair\overset{h\upsilon }{⟶}electron\;+\;hole$. If the light asymmetrically illuminates the membrane, the photo-excited electrons and holes will diffuse from the illuminated area to the non-illuminated part. In an open circuit state, electrons and holes will finally establish a new electric potential difference among the GO membrane [36].

Three different models were considered, as shown in Figure 1. The light illuminated from left to right with an illuminating window width of 5 mm, consistent with previous experimental conditions [36]. When light is illuminated on the GO membrane, the voltage of the corresponding area was lowered.

We developed a one-dimensional continuum model to describe the illumination process. Considering the items of photo-excited emergence, recombination, diffusion, and electric-field-induced migration of carriers, we have the diffusion functions shown in Equation (1).

(1) $$\begin{array}{c} \frac{\partial \rho_{e}\left(x,t\right)}{\partial t}=gW\left(x,t\right)-h\rho_{e}\left(x,t\right)\rho_{h}\left(x,t\right)+D_{e}\frac{\partial^{2}\rho_{e}\left(x,t\right)}{\partial x^{2}}-\mu_{e}\frac{\partial \left[\rho_{e}\left(x,t\right)\nabla \psi \left(x,t\right)\right]}{\partial x}\\ \frac{\partial \rho_{h}\left(x,t\right)}{\partial t}=gW\left(x,t\right)-h\rho_{e}\left(x,t\right)\rho_{h}\left(x,t\right)+D_{h}\frac{\partial^{2}\rho_{h}\left(x,t\right)}{\partial x^{2}}+\mu_{h}\frac{\partial \left[\rho_{h}\left(x,t\right)\nabla \psi \left(x,t\right)\right]}{\partial x} \end{array}$$

where the subscripts *e* and *h* represent electron and hole, respectively. $\rho \left(x,t\right)$ is the local carrier density, $\psi \left(x,t\right)$ is the local electric potential, *g* is the photoresponsivity, $W\left(x,t\right)$ is the incident light intensity at a specific position *x*, *h* is the recombination coefficient, *D* is the carrier’s diffusion coefficient, and μ is the carrier’s electric mobility.

From Einstein relation, we can get the electric mobility μ as follow:

(2) $$\begin{array}{c} \mu =\frac{2eD}{k_{B}T_{e}} \end{array}$$

where *e* is elementary charge, $k_{B}$ is Boltzmann constant, $T_{e}$ is carrier temperature, here $T_{e}=3600\;K$ [39]. According to Equations (1) and (2), there are five independent parameters, i.e., $g,W,h,D_{e},$ and $D_{h}$.

According to boundary condition, as shown in Equation (3), there is no net electron or hole migration at two ends of the board, which means concentration diffusion equals electric migration at two ends.

(3) $$\begin{array}{c} D_{e}\frac{\partial \rho_{e}\left(x,t\right)}{\partial x}-\mu_{e}\rho_{e}\left(x,t\right))\nabla \psi \left(x,t\right)=0\\ D_{h}\frac{\partial \rho_{h}\left(x,t\right)}{\partial x}+\mu_{h}\rho_{h}\left(x,t\right))\nabla \psi \left(x,t\right)=0 \end{array}$$

We use Poisson’s Equation to solve the last terms of Equation (3), as shown in Equation (4).

(4) $$\begin{array}{c} -\nabla^{2}\psi \left(x,t\right)=\frac{\rho }{\epsilon } \end{array}$$

According to the state-of-art references, the traditional parameters mentioned above are listed in Table 1. The values are the good starting points to study these parameters’ effect on photo-induced fast transport of ions in 2D nanochannels.

Use numerical integration to calculate Equation (1) with the time step of 0.1 ps. The initial linear densities of carries are set to $1.0\times 10^{-9}\;\mathrm{mol}/m$. The electric potential at the beginning is 0 V. The length of the graphene-oxide membrane model is 15 mm, according to the related experiments. The illuminated length is 5 mm, 1/3 of the total length [36].

### 2.2. Model for Ion Transport Inside the Nanochannel

Figure 2 gives the ion transport model inside nanochannel, used for MD simulation to give the ionic current induced by light.

MD simulation was conducted using the NAMD package [45], and the visualizations were represented by VMD [44]. The geometry of the lamellar nanochannel was 10.0nm(length)×1.3nm(height)×3.3nm(width) which was connected to two reservoirs sized by 4.6nm(length)×5.4nm(height)×3.3nm(width).

The template simulation box has the same size as the nanochannel structure and the carbon atom array, which is shown in Figure 2, is built with the same center as the template simulation box to mimic real GOM. SOLVATE script in VMD is used to create a solvation environment and pretreat carbon atoms structure. After that, all water molecules are deleted and carbon atoms are fixed. Then simulation box sized 14.5nm(length)×7.4nm(height)×5.4nm(width) is created and a nanochannel frame is put to box center. Again, SOLVATE script is used to solvate the whole box with water molecules under 1.0g/mL and a pretreatment simulation in 2ns with NPT ensemble is run under 1atm, 300K. Last frame of pretreatment result file is dragged out and deleted unnecessary water molecules (two space above and under the nanochannel in Figure 2). This is how the initial simulation box is created.

For each surface charge, only carbon atoms consisting of the channel part (not include the wall carbon atoms of reservoirs) will be charged. Then, AUTOIONIZE script in VMD is used to add ions and neutralize the box. After that, 1mol/LKCl is generated in water. Here the reservoirs offering solution volume to decrease the difference counts of $K^{+}$ and ${\mathrm{Cl}}^{-}$ due to the surface charge. In the simulation, when the surface charge is $-17.2\;\mathrm{mC}/m^{2}$, there are 74 $K^{+}$ atoms and 67 ${\mathrm{Cl}}^{-}$ atoms. When the surface charge is $-34.4\;\mathrm{mC}/m^{2}$, there are 81 $K^{+}$ atoms and 67 ${\mathrm{Cl}}^{-}$ atoms.

The simulation procedure is similar to our previous work [46,47,48]. The all-atoms charmm22 force field was briefly used to describe bonded and non-bonded interactions among atoms [49]. SPC/E model was used for water molecules. The cut-off distances for both L-J interaction and Long-range electrostatic interactions (via the particle mesh Ewald method, PME) were set to 1.4nm. The carbon atoms were fixed to their initial position. Electrical field was added along the length (*x*) axis to simulate the voltage difference induced by illumination, ranging from 0 up to $0.4\;\mathrm{kcal}/\mathrm{mol}\cdot \overset{{}^{\circ}}{A}\cdot e$. The SETTLE algorithm maintained the geometry of water molecules. For time integration, a leapfrog algorithm with a time step of 1.0fs was used. The recording frequency of calculated trajectories was set to 500steps, 0.5ps. System temperature maintained at 300K by applying a dual Langevin thermostat with 5.0ps in Langevin damping coefficient. The periodic boundary condition is set on length and width direction in Figure 2.

For each simulation in canonical (NVT) ensemble of given surface charge density and electric field, the data of 33ns was segmented into four parts. The beginning 3.0ns was for re-equilibrium, which was obsoleted. For the rest 30.0ns, every 10.0ns was considered an independent parallel experiment. We counted ions that go through the channel at three different positions for each second. For an individual experiment, three positions were calculated using mean ion flow, and total mean flux and standard deviation were calculated using three parallel results.

## 3. Results and Discussion

### 3.1. The Effect of Illuminate Position on the Voltage Difference

**Figure 3:**
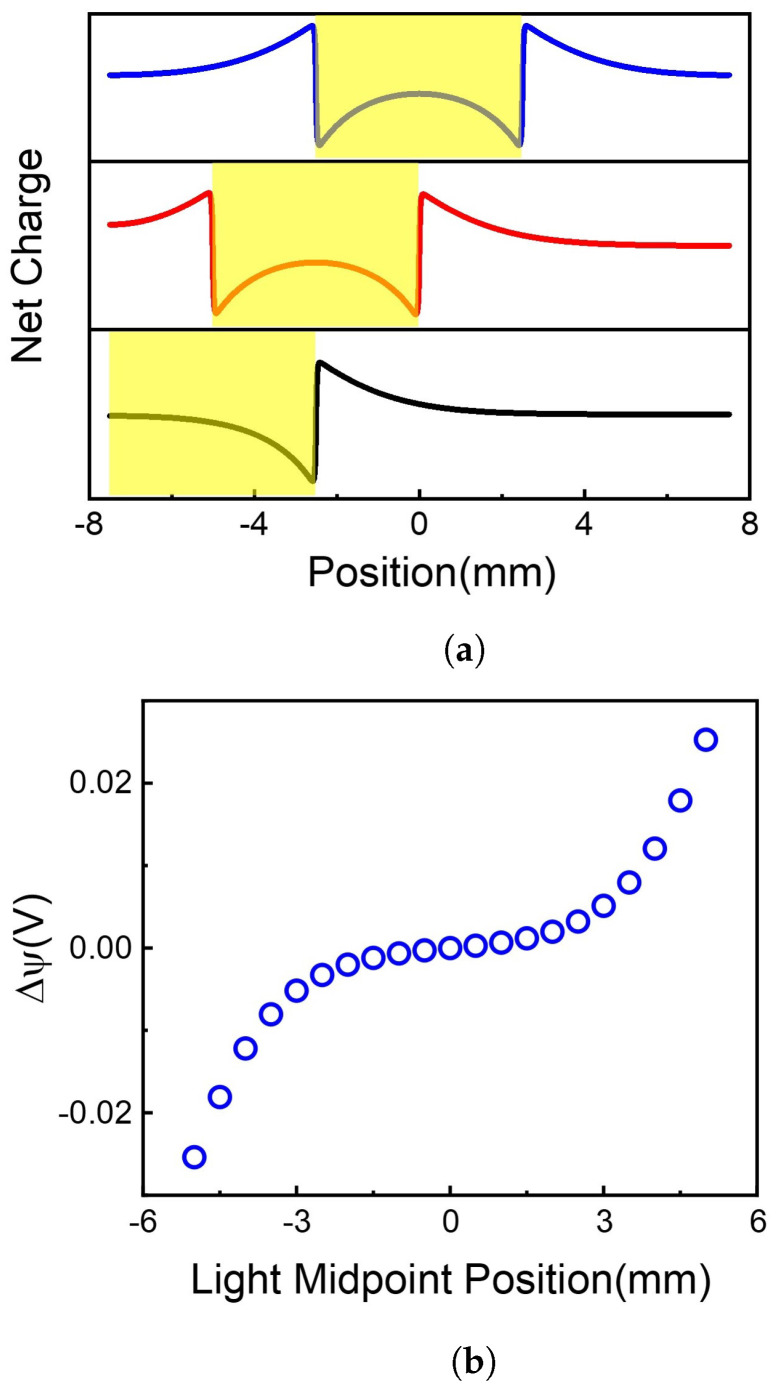
Net charge distribution and voltage difference along 2D membrane at different illumination center positions. (**a**) Light illumination causes the charge redistribution along the 2D membrane. (**b**) The voltage difference caused by different illumination center position.

We first studied the effect of illumination position on the voltage difference between two terminals of the 2D membrane, as shown in Figure 3. Here the length of the 2D membrane is 15 mm, and the illuminated width is fixed at 5 mm. The light beam is centered in the range of –5.0 to 5.0 mm along the *x*-direction with a space of 0.5 mm.

By solving Equation (1) at different initial values of W, the net charge distributions were obtained, as shown in Figure 3a. The centers of illumination are located at x=−5.0,−2.5 and 0.0mm, respectively. It is shown that although the total net charges remain neutral on the 2D membrane, the net charge distributions are highly position-dependent. When the light illuminates on the center of the 2D membrane (x=0.0mm), the area of light-illumination has some negative charges, resulting in a sharp increase of positive charges at the edge of light-illumination, i.e., x=−2.5 and 2.5mm. The positive charge gradually decays to a minimum at the terminals of the 2D membrane. Because there is symmetric net charge distribution on the 2D membrane, no voltage difference occurs between the two ends of the membrane, as shown in Figure 3b (x=0mm).

When the light illuminates asymmetrically on the 2D membrane, e.g., at x=−5.0mm, the charges at the two ends of the 2D membrane are different, resulting in the voltage difference shown in Figure 3b (x=−5.0mm). Therefore, asymmetric illumination is the reason for the voltage difference between the two ends of the membrane. The voltage difference highly depends on illumination position, as shown in Figure 3b. More asymmetrically illuminated, more net charge difference, resulting in higher voltage difference.

We chose x=−5.0mm the illumination center position in further calculations and discussions.

### 3.2. The Effect of Photoresponsivity on the Voltage Difference

**Figure 4:**
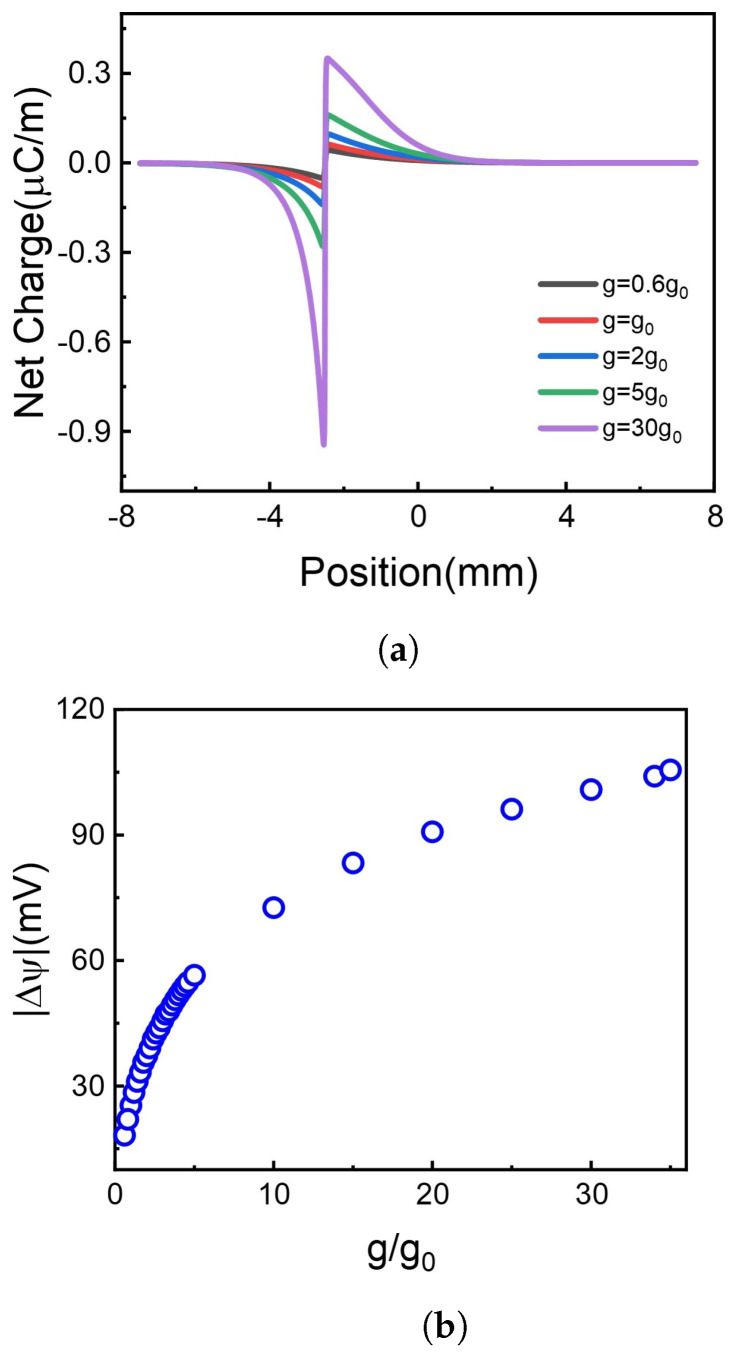
The net charge distribution and the corresponding voltage difference at different photoresponsivity. (**a**) Net charge distribution. (**b**) Voltage difference.

The formation of hole and electron is the source of free carriers, highly dependent on the photoresponsivity, *g*. The reference photoresponsivity ($g_{0}$) is 45 mA/W, which is obtained from reference [40]. Here we changed values of *g* from $0.6\;g_{0}$ to $30\;g_{0}$. The effect of the g value on the net charge distribution and the absolute potential difference is given in Figure 4.

It is shown in Figure 4a that net charges distinctly accumulate at the borderline of illuminate and non-illuminate. The increase of g leads to more net charge accumulation at the borderline. Although the net charge decays gradually along the 2D membrane, the accumulated net charge results in the voltage difference, as shown in Figure 4b. Higher photoresponsivity enriches more net charge accumulation, bringing more significant potential difference. As shown in Figure 4b, the absolute value of voltage difference between two terminals of the 2D membrane increases monotonously with photoresponsivity.

Hence, raising the photoresponsivity of graphene oxide improves the voltage difference along the membrane, in line with recent experimental observation. For example, Kang et al. fabricated enhanced and wavelength-selective photoresponsivity graphene photodetector by crumping [50], offering more graphene surface per unit area. The limit of graphene-based material photoresponsivity is weak optical absorption [38]. Gan et al. reported that graphene doped with metal could reach a photoresponsivity exceeding 100 mA/W [51]. Also, one can exfoliate heterostructures exploiting the broad-band transparency of graphene and a special bandgap of other materials. Mudd et al. reported InSe-Graphene van der Waals heterostructures with high broad-band photoresponsivity and wider spectrum response range from near-infrared to visible spectrum [52].

### 3.3. The Effect of Recombination Coefficient on the Voltage Difference

**Figure 5:**
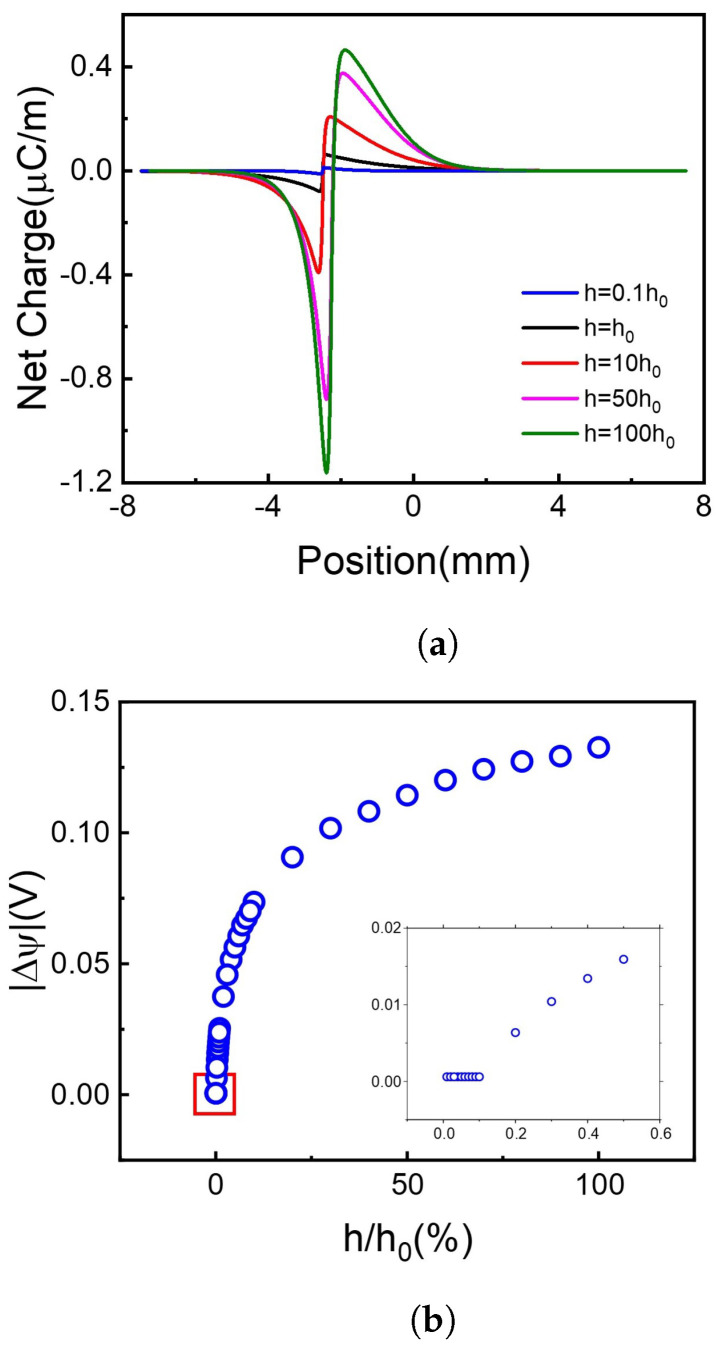
The net charge distribution and absolute voltage difference at different recombination coefficients. (**a**) Net charge distribution. (**b**) Voltage difference.

Another important parameter is the recombination coefficient, *h*, which gives the recombination rate of the carriers (holes and electrons). A higher value of *h* facilitates the recombination of carriers. Here we calculated the effect of relative recombination coefficient, $h/h_{0}$, on the net charge distribution and voltage difference, as shown in Figure 5.

It is shown in Figure 5a that when the *h* value increases, meaning that more holes and electrons recombine with each other, more net charges accumulate on the borderline of the illuminated and non-illuminated area of the 2D membrane. It is out of our initial guess and greatly increases our interest!

Firstly, we focus on the net charge distribution in Figure 5a. Supposing now is a steady-state, the board is separated into two regions, light region, and dark region. In unit time, both types of carriers migrate from light region to dark region obey Fick’s law, $F_{flux}=D_{diff}\times \left(C_{light}-C_{dark}\right)$, *F* is the flux of proton or electron, *D* is diffusion coefficient, *C* is steady carrier concentration. On one hand, illuminating area generates abundant carriers which are not influenced by recombination, thus $C_{light}$ would not change along with recombinant coefficient. On the other hand, recombination dominant $C_{dark}$ means $C_{dark}$ decrease with a higher recombinant coefficient. This leads to the increase of $\left(C_{light}-C_{dark}\right)$ when the recombinant coefficient increases. Thus, due to the difference of Ddiff of hole and electron, when recombinant coefficient increases, the net charge flux, $F^{net}=F^{proton}-F^{electron}$ also increases. That’s the reason why the net charge increases with the recombinant coefficient increase (Figure 5a), leading to the voltage difference more significant in Figure 5b.

Secondly, the subplot in Figure 5b shows that the voltage difference remains to a very small amount above 0 when the recombination coefficient continuously decreases. Here we investigated the carriers’ distribution. The carriers’ concentration increases with the recombination coefficient decreasing, which agrees with the former conclusion. But due to the calculation error, we cannot get enough accurate data with tiny *h*. For more information, please see Supporting Information Figure S1.

To draw a brief conclusion, choosing materials with a higher recombination coefficient can significantly increase the voltage difference in range $h/h_{0}\in \left[0.5,25\right]$. Further increase of recombination coefficient will have a limited effect on the voltage difference.

### 3.4. The Effect of $D_{e}$ and $D_{h}$ on the Voltage Difference

**Figure 6:**
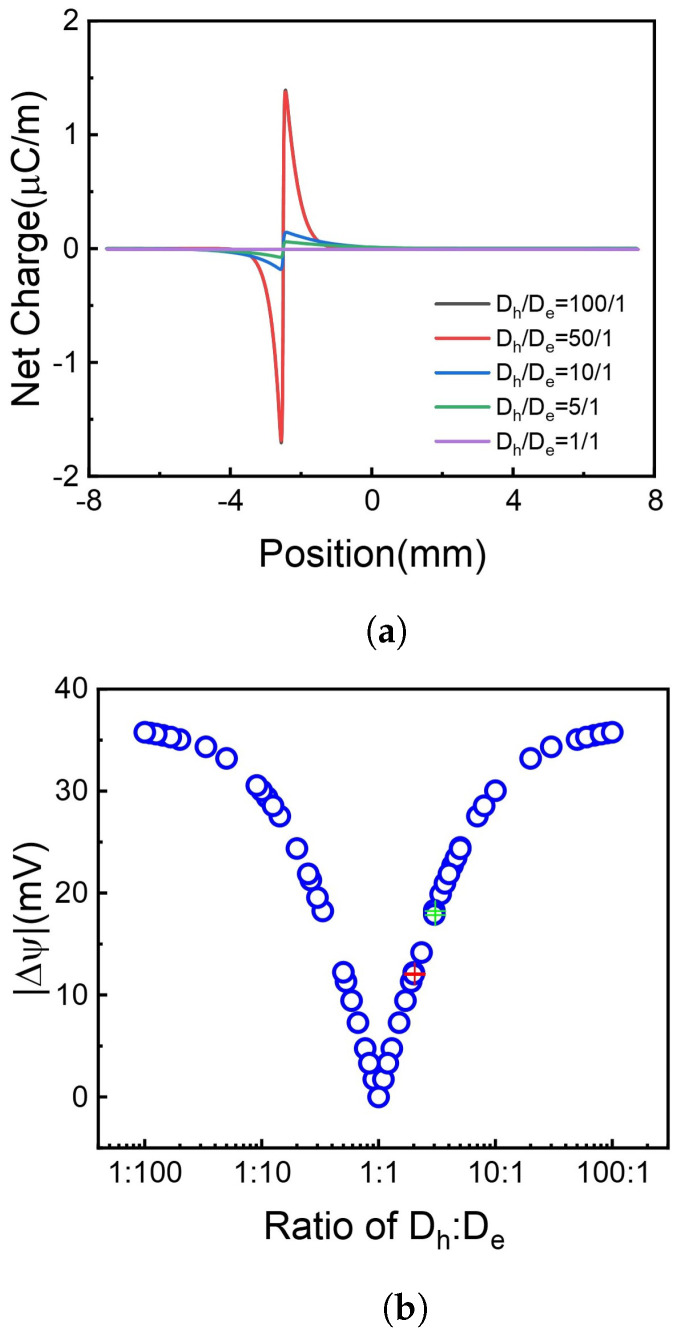
The net charge distribution and absolute voltage difference at different $D_{e}/D_{h}$ ratios. (**a**) Net charge distribution. (**b**) Voltage difference, red and green cross stands for same ratio but different absolute value. Different colors indicate different values but the same ratio.

It is shown in Figure 6 that when the divergence of $D_{e}$ and $D_{h}$ increases, meaning the diffusion of holes and electrons is more asymmetrical, the voltage difference increases. Furthermore, the rise of voltage difference becomes inconspicuous when the diffusion coefficients become extremely inequality, e.g., $D_{h}/D_{e}>50:1$ or $D_{h}/D_{e}<1:50$. Compared to the ratio of two diffusion coefficients, the absolute numerical value has little effect on the generation of the voltage difference. Some situations are calculated with the same diffusion coefficient ratio but different values, shown in Figure 6b red and green cross symbols, as a point set which gathers together, e.g., the voltage difference has only a little relation with the diffusion coefficient value.

For the same ratio but different absolute numerical values, which is the red and green cross in Figure 6b please see Supporting Information Figure S2.

### 3.5. Photocurrent Induced by Illumination

**Figure 7:**
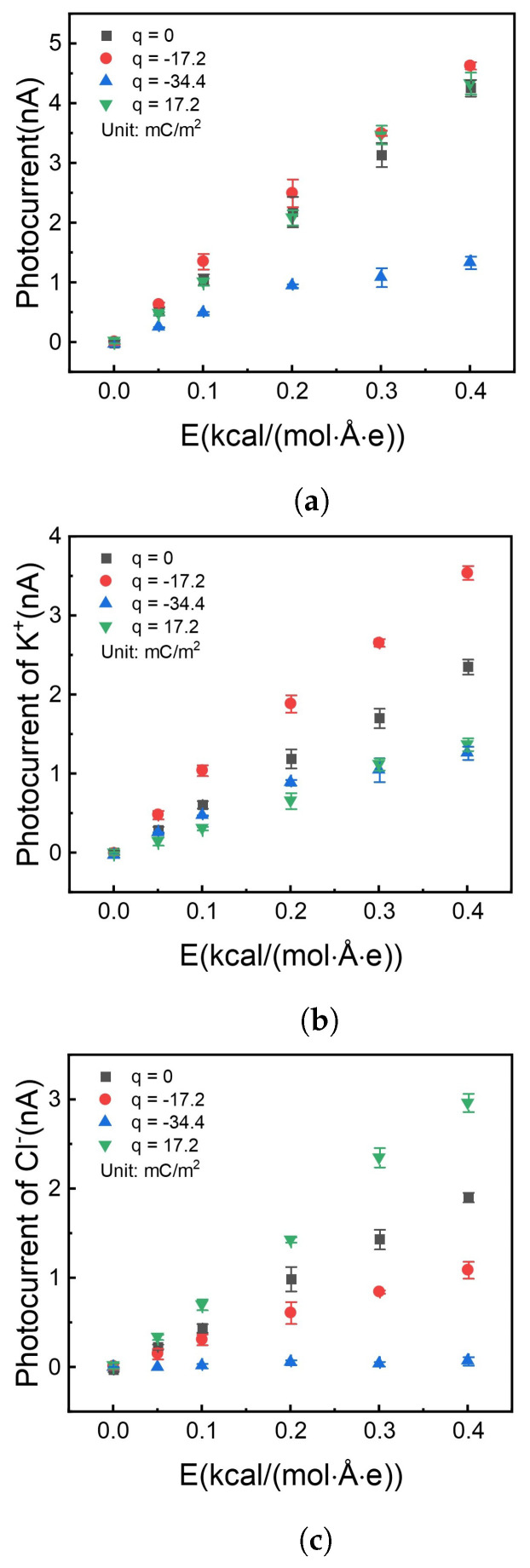
Photocurrent vs. different electrical fields with different charge densities on the internal surface of the nanochannel. (**a**) Total photocurrent. (**b**) Photocurrent offered by potassium ion. (**c**) Photocurrent offered by chloride ion.

Count the ion flux at three cross-sections, which were perpendicular to the x-axis in a nanochannel, located at the right middle of the channel and ±3.7nm. The mean of three counts was the flux at a specific case. The stand deviation was calculated as an error bar. All the results were shown in Figure 7.

It is shown in Figure 7a that the photocurrent has no apparent difference with surface charge density at 0, −17.2, and +17.2 $\mathrm{mC}/m^{2}$. But Figure 7b,c show the difference in the contribution of different types of ions to the photocurrent. Firstly, when the surface is not charged (black square symbol), the potassium ion has more current contribution (flux rate) than the chloride ion due to the size-dependent properties. Potassium ion has a smaller diameter, which tends to get through the nanochannel much easier.

Secondly, when the channel surface was doping with charge, the ions with anti-charge play the most important part in the current flow. The difference in diffusion can be attributed to the ability for active ion sieving. By charging the surface with a negative charge, $K^{+}$ can be effectively pumped over three times than ${\mathrm{Cl}}^{-}$ (red circle marks in Figure 7b,c) with the same total photocurrent as the uncharged situation (Figure 7a). This phenomenon was due to the electrostatic repulsion between the surface charge and ions.

When surface charge density increases to $-34.4\;\mathrm{mC}/m^{2}$, the photocurrent reduces in Figure 7a (blue symbol). On the one hand, enhanced surface charge thickens the electrical double layer [53], which reduces the effective channel height. On the other hand, a negative surface charge prevents chloride ions from getting into the nanochannel. The above two reasons result in that only potassium ion can get through the nanochannel, and photocurrent reduces than those with less surface charge density.

## 4. Conclusions

In this work, we build a light-induced carriers’ diffusion model basing on our previous experimental results. This model can reveal the light-induced ion transport mechanism and predict the ion behavior under different operational situations and material characters. We find that illustrate position affects the asymmetry of charge distribution, which causes the voltage difference between the two board sides with a positive correlation. Choose the material with higher photoresponsivity will also enhance the charge distribution asymmetry, bringing a larger voltage difference. However, a higher recombination coefficient also increases voltage difference, which is not intuitionistic. Here we study absolute charge distributions and propose a plausible diffusion constitutive equation, like Fick’s law. It is shown that a higher recombination coefficient indeed decreases two types of charge distribution, whereas the net charge distribution increases due to diffusion coefficient difference, which enriches the voltage difference. According to our carriers’ diffusion model, two types of charge migration depends on their diffusion coefficient. Thus, net charge distribution is strongly affected by the diffusion coefficient ratio. The diffusion ability is more different when the voltage difference is higher. Furthermore, the absolute numerical number of diffusion coefficients has no significant effect on voltage difference with the same ratio. We can conclude that illuminate asymmetry and diffusion process decide voltage difference. We build up a simulation box based on voltage difference to study the ion transport behavior along with different nanochannel surface charges with molecular simulation. We find that moderate surface charge decreases the ion transport with the same type of charge due to the electrostatic repulsion. However, excess surface charge blocks both cation and anion because a thicker electrical double layer decreases effective channel height.

## Figures and Tables

**Table 1:** Parameters for carriers’ diffusion model calculation.

Parameters	Values	References
Photoresponsivity (*g*)	45mA/W	Ref. [40]
Recombination coefficient (*h*)	$2.6\times 10^{12}\;m^{2}/\left(\mathrm{mol}\cdot s\right)$	Refs. [41,42]
Hole mobility ($\mu_{h}$)	$3.4\times 10^{4}\;{\mathrm{cm}}^{2}/\left(V\cdot s\right)$	Ref. [39]
Electron mobility ($\mu_{e}$)	$6.8\times 10^{3}\;{\mathrm{cm}}^{2}/\left(V\cdot s\right)$	Refs. [41,43]
Diffusion coefficient of hole ($D_{h}$)	$5.5\times 10^{3}\;{\mathrm{cm}}^{2}/s$	Equation (2)
Diffusion coefficient of electron ($D_{e}$)	$1.1\times 10^{3}\;{\mathrm{cm}}^{2}/s$	Equation (2)

## Supplementary Materials

The following are available online, Figure S1: The net charge, hole, and electron distribution, Figure S2: The net charge distribution at different diffusion coefficient values with the same ratio, Figure S3: MD simulation with a larger reservoir (10 nm in length), Figure S4: MD simulation under a long time(100 ns), Figure S5: Ion concentrations in reservoir and channel after 100ns simulation.
