# Supplementary material for: Ionic Transport Triggered by Asymmetric Illumination on 2D Nano-Membrane
Source: Molecules. 2021 Nov 23;26(23):7078. doi: 10.3390/molecules26237078 (PMC8658790; doi:10.3390/molecules26237078)
Supplement: Supplementary file 1 [file molecules-26-07078-s001.zip › molecules-1460506-supplementary.pdf]

Supporting information

SI.1 Carriers' concentration distribution at the difference *h*

Due to numerical integration, we cannot get notable net charge distribution when *h* goes small. Still, the carriers' distribution does increase with *h* decreases.

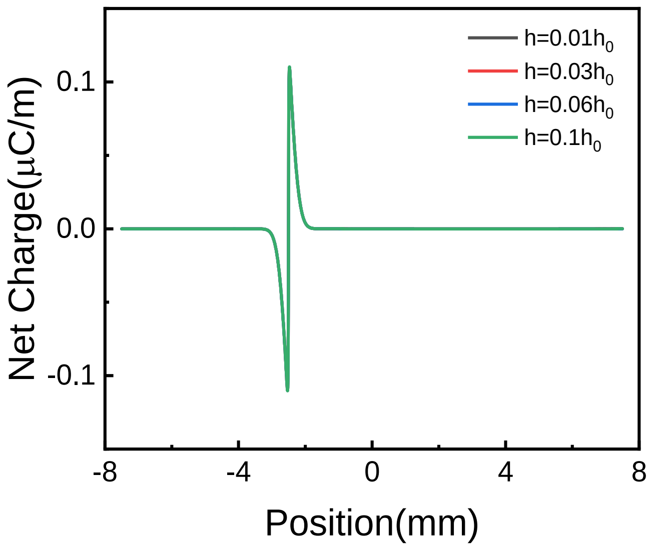

(a) Net charge distribution

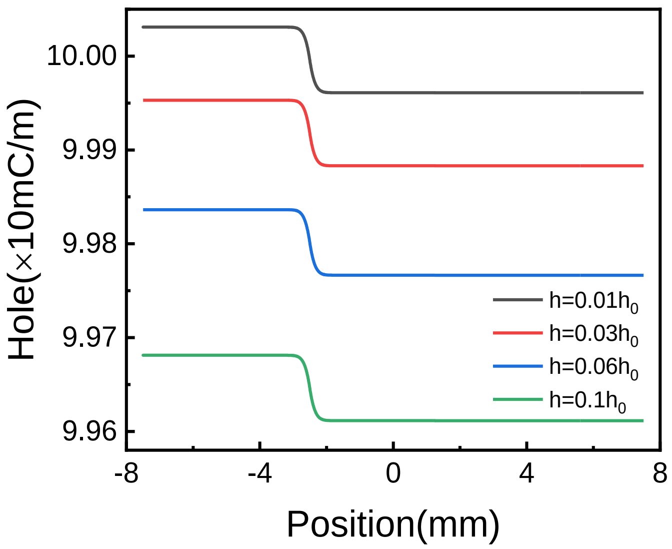

(b) Hole distribution

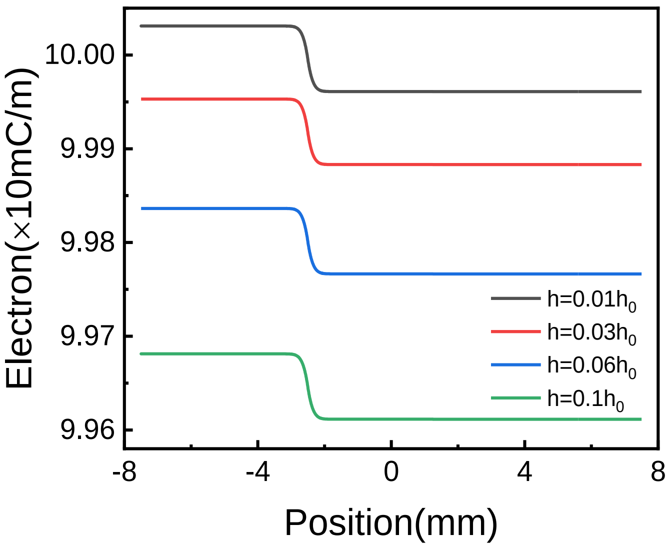

(c) Electron distribution

Figure S1. The net charge, hole, and electron distribution.

SI.2 Same ratio but the different value of D_h/D_e

Fig. S2 shows an unexpected net charge distribution, which has only a little effect on voltage distribution. To explain the results, we should go back to Eq. (1). The item $gW(x,t)$ is the source of carriers, which was fixed to a constant. Raising the parameters of the item relating to diffusion coefficients $D\&\mu$ but keep the recombination coefficient has the same result to decrease the recombination coefficient but keep the same diffusion coefficients. This is only a numerical trick on Eq. (1). The discussion here can be referred to section *The effect of recombination coefficient on the voltage difference*.

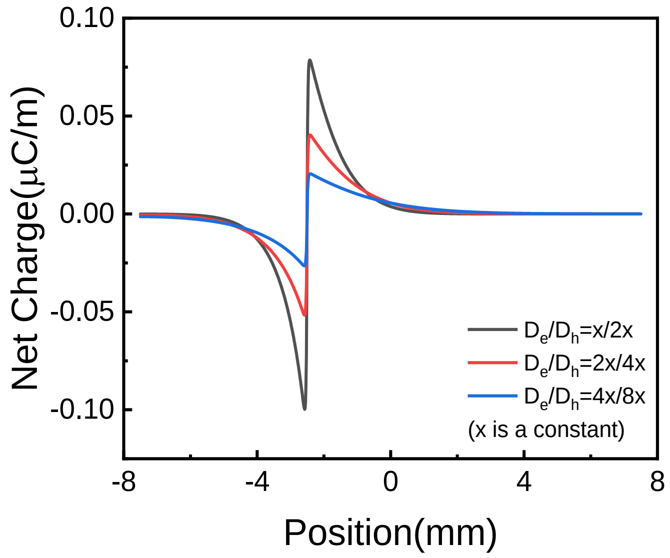

Figure S2. The net charge distribution at different diffusion coefficient values with the same ratio.

SI.3 Larger reservoir

To estimate the influence of different reservoir sizes, we conducted an MD simulation with a larger reservoir, which is 2.28 times the original model in the main text. Surface charge is –17.2 mC/m^2, electric field is 0.3 kcal/(mol×Å×e). The results showed that increasing the electrolyte solution reservoir did not change the simulation results, as shown in the figure below. The current contribution of K^+ is 546, which for Cl^- is 204 (in total 33ns).

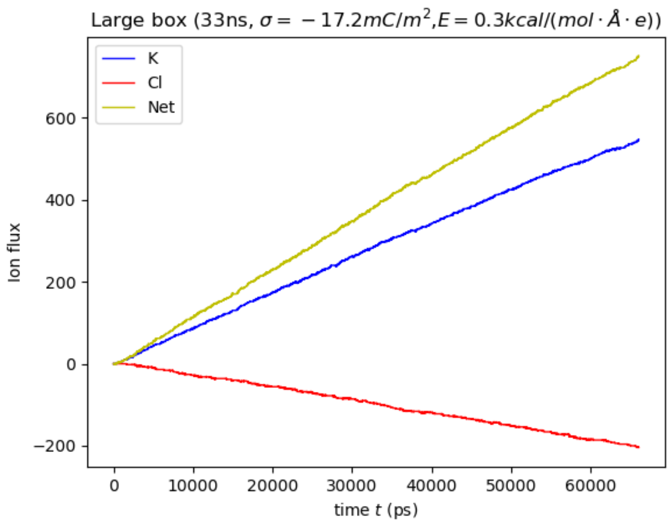

Figure S3. MD simulation with a larger reservoir (10 nm in length).

SI.4 Long time simulation

We conducted the MD simulation for 100ns with a surface charge of –17.2 mC/m^2 and electric field 0.3 kcal/(mol×Å×e). It is shown that 30 ns is long enough for statistical analysis.

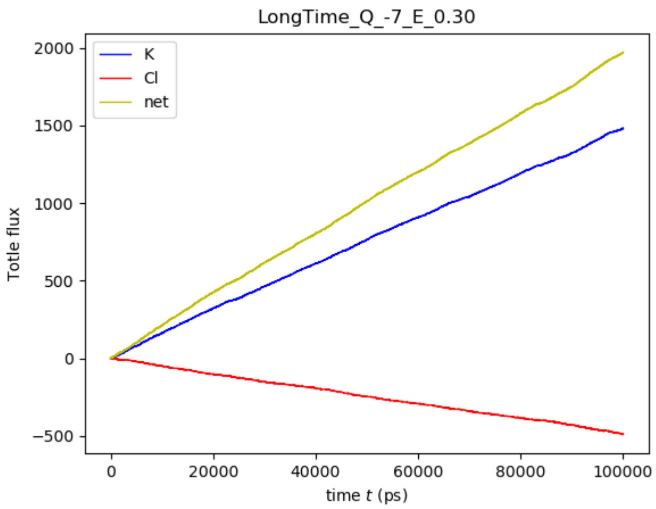

Figure S4. MD simulation under a long time(100ns).

SI.5 Temperature effect

We have estimated temperature increase due to illumination power in our previous work's Supplementary information (https://static-content.springer.com/esm/art%3A10.1038%2Fs41467-019-09178-x/MediaObjects/41467_2019_9178_MOESM1_ESM.pdf).

In Supplementary Note4. Temperature effect (page 8), we measured the highest temperature increase due to illumination firstly, then we established a local high-temperature area on the GO strip by a ceramic heater to simulate the photothermal effect and checked the influence of the temperature on the ion transport property. During heating the GO, no current was found. Also, in our experiments, the temperature rise was typically less than 20℃. Therefore, the thermoelectric property is negligible. After careful consideration, we judged that the temperature effect is insignificant at conditions considered in this work.

SI.6 Ion concentration in nano-channel and reservoir

We analyzed the situation in SI.4 Long time simulation. We have:

The mean Cl^- in the channel is 0.445 mol/L

The mean Cl^- in the reservoir is 1.129 mol/L

The mean K^+ in the channel is 0.662 mol/L

The mean K^+ in the reservoir is 1.159 mol/L

The corresponding figure is given below.

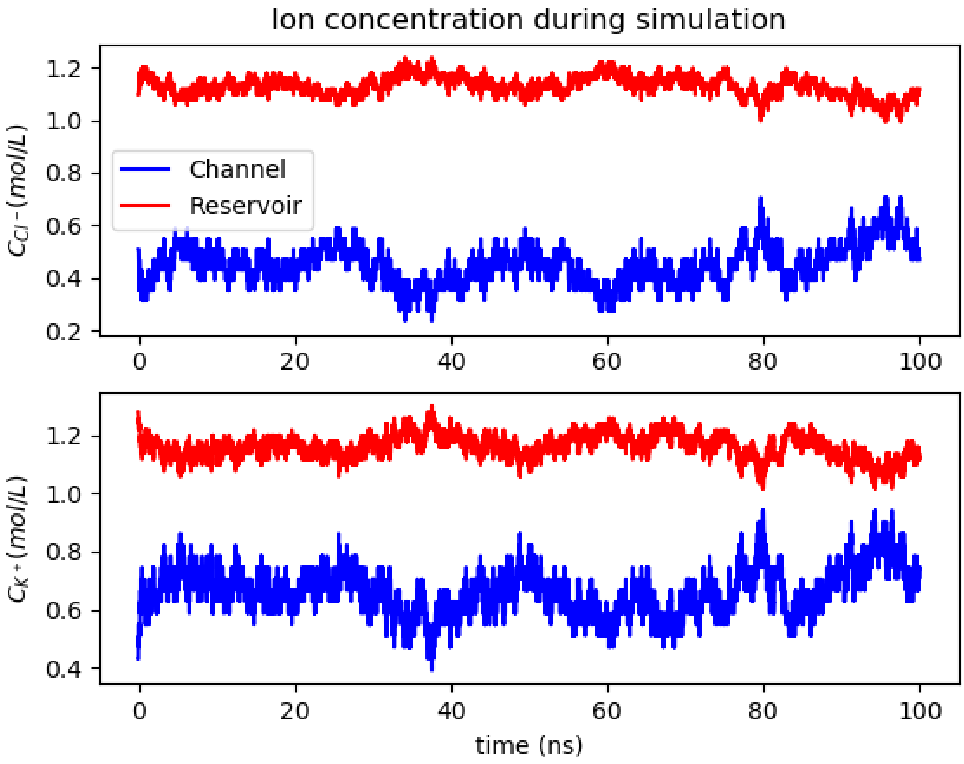

Figure S5. Ion concentrations in reservoir and channel after 100ns simulation
